# Predicting In-hospital Mortality Using D-Dimer in COVID-19 Patients With Acute Ischemic Stroke

**DOI:** 10.3389/fneur.2021.702927

**Published:** 2021-07-16

**Authors:** Youngran Kim, Swapnil Khose, Rania Abdelkhaleq, Sergio Salazar-Marioni, Guo-Qiang Zhang, Sunil A. Sheth

**Affiliations:** Department of Neurology, UTHealth McGovern Medical School, Houston, TX, United States

**Keywords:** D-dimer, COVID-19, stroke, mortality, coagulopathy, electronic medical records, coronavirus

## Abstract

**Background:** Coronavirus disease 2019 (COVID-19) has been associated with coagulopathy, and D-dimer levels have been used to predict disease severity. However, the role of D-dimer in predicting mortality in COVID-19 patients with acute ischemic stroke (AIS) remains incompletely characterized.

**Methods:** We conducted a retrospective cohort study using the Optum® de-identified COVID-19 Electronic Health Record dataset. Patients were included if they were 18 or older, had been hospitalized within 7 days of confirmed COVID-19 positivity from March 1, 2020 to November 30, 2020. We determined the optimal threshold of D-dimer to predict in-hospital mortality and compared risks of in-hospital mortality between patients with D-dimer levels below and above the cutoff. Risk ratios (RRs) were estimated adjusting for baseline characteristics and clinical variables.

**Results:** Among 15,250 patients hospitalized with COVID-19 positivity, 285 presented with AIS at admission (2%). Patients with AIS were older [70 (60–79) vs. 64 (52–75), *p* < 0.001] and had greater D-dimer levels at admission [1.42 (0.76–3.96) vs. 0.94 (0.55–1.81) μg/ml FEU, *p* < 0.001]. Peak D-dimer level was a good predictor of in-hospital mortality among all patients [c-statistic 0.774 (95% CI 0.764–0.784)] and among patients with AIS [c-statistic 0.751 (95% CI 0.691–0.810)]. Among AIS patients, the optimum cutoff was identified at 5.15 μg/ml FEU with 73% sensitivity and 69% specificity. Elevated peak D-dimer level above this cut-off was associated with almost 3 times increased mortality [adjusted RR 2.89 (95% CI 1.87–4.47), *p* < 0.001].

**Conclusions:** COVID-19 patients with AIS present with greater D-dimer levels. Thresholds for outcomes prognostication should be higher in this population.

## Introduction

Coronavirus disease 2019 (COVID-19) caused by severe acute respiratory syndrome Coronavirus-2 (SARS-CoV-2) is primarily a respiratory tract infection, but coagulopathy associated with its profound inflammatory response has been well-described (1–3). D-dimer, a degradation product of cross-linked fibrin that reflects ongoing activation of the coagulation cascade, has been linked with coagulopathy in COVID-19 infection. Elevated D-dimer level has been identified as a useful

predictor for mortality in patients with COVID-19 and several studies demonstrated its prognostic potential and optimal cutoff value (4–6). However, the prognostic value of D-dimer in predicting COVID-19 mortality has been tested mostly from single provider or pooled meta-analyses (4–7), and the performance and optimal cutoff value in patients with acute ischemic stroke (AIS), a condition that may independently elevate D-dimer (8, 9), remains uncharacterized. Here, we examine whether D-dimer remains useful to predict mortality in COVID-19 patients identified from a large multicenter sample and determine the optimal cutoff value to predict mortality in COVID-19 patients presenting with AIS. We study a broad time period including more recent COVID-19 cases and cover national level geographic regions to include multiple COVID-19 pandemic surges and viral strains.

## Methods

### Data Source

We conducted a retrospective cohort study using the Optum® de-identified COVID-19 Electronic Health Record (EHR) dataset. Given the urgent need to clinically understand the novel virus of COVID 19, Optum developed a data pipeline that enables minimal data lag, while preserving as much clinical data as possible. The data is sourced from Optum's longitudinal EHR repository, which is derived from dozens of healthcare provider organizations in the United States, which include more than 700 hospitals and 7,000 clinics across the continuum of care. The COVID-19 dataset incorporates a wide swath of raw clinical data, including new, unmapped COVID-specific clinical data points from both Inpatient and Ambulatory electronic medical records, practice management systems, and numerous other internal systems. The Optum COVID-19 data elements include demographics, mortality, diagnoses, procedures, medications prescribed and administered, lab results, and other observable measurements.

### Study Population

Patients were included if they had laboratory-confirmed COVID-19 between March 1, 2020, and November 30, 2020 (*n* = 281,665) and were hospitalized within 7 days of the positivity date (*n* = 35,919). Positive COVID-19 status was determined by the detection of SARS-CoV-2 in polymerase chain reaction (PCR) test, and the positivity date was based on the date of sample collected. We limited the study to individuals who were tested and had valid results for D-dimer at admission or during their hospitalization (*n* = 15,313). Patients who were younger than 18 years (*n* = 52) or had missing sex information (*n* = 11) were excluded.

### Measurements of D-dimer and Other Variables

D-dimer values within 24 h of admission and the peak values recorded during hospital stay were tested to predict all-cause mortality during the index COVID-19 hospital stay. Because the data was sourced from multiple laboratories, D-dimer results varied in reporting units. D-dimer results can be reported using a fibrinogen equivalent unit (FEU) or using a D-dimer unit (DDU) depending on the molecular weight used. FEU reports D-dimer levels based on the molecular weight of fibrinogen, whereas DDU reports D-dimer levels based on its own molecular weight, which is about half that of fibrinogen. We approximated D-dimer levels reported in DDU to those in FEU by multiplying by 2. AIS was identified using the International Classification of Diseases, Ninth Revision, Clinical Modification (ICD-9-CM) codes (433.x1, 434.x1 and 436) and ICD-10-CM code (I63.x). Although ICD-9-CM codes have been replaced with ICD-10-CM as of October 2015, ICD-9 codes were included to identify AIS and comorbidities as some patient medical records were still reported using ICD-9 codes. We also examined medication usages, particularly antiplatelets (aspirin, clopidogrel, prasugrel, ticagrelor, dipyridamole, and eptifibatide) and anticoagulants given during hospitalization.

### Statistical Analysis

The optimal D-dimer cutoff point and C-statistic of D-dimer levels were evaluated by receiver operator characteristic (ROC) curve for all COVID-positive patients and a subgroup of patients with AIS at admission. The probability of survival during the hospital stay was plotted for patients above and below the cutoff level of D-dimer using Kaplan-Meier survival functions, and the difference in survival curves was assessed using log-rank test. The prognostic value of D-dimer was assessed using a modified Poisson regression model, and risk ratio (RR) was estimated adjusting for age, sex, race/ethnicity, known risk factors, and medication use (10). Significance levels were set at *P* < 0.05 for 2-tailed tests and all analyses were performed using STATA 16.0 (StataCorp, College Station, TX).

## Results

Among 15,250 patients hospitalized with COVID-19 positivity, 285 presented with AIS at admission (2%). Patients with AIS were older [median age 70 (60–79) vs. 64 (52–75)] and had higher prevalence of congestive heart failure, hypertension, diabetes, vascular disease, and atrial fibrillation. D-dimer levels at admission were greater for patients presenting with AIS [median (IQR), 1.42 (0.76–3.96) μg/ml FEU] compared to those without AIS [0.94 (IQR 0.55–1.81) μg/ml FEU] and peak levels were also greater for patients with AIS [3.86 (IQR 1.23–15.58) vs. 1.42 (IQR 0.76–3.96) μg/ml FEU]. All other lab values on admission were similar between patients with and without AIS except neutrophil and white blood cell counts in AIS patients (Table 1).

**Table 1:** Characteristics of patients in COVID with or without acute ischemic stroke.

****	**Total (*N* = 15,250)**	**No AIS at admission (*n* = 14,965)**	**AIS at admission (*n* = 285)**	***p*-value**
Age, median (IQR)	64 (52–75)	64 (52–75)	70 (60–79)	<0.001
Age ≥ 65, *n* (%)	7,525 (49.3)	7,340 (49.0)	185 (64.9)	<0.001
Male, *n* (%)	8,371 (54.9)	8,199 (54.8)	172 (60.4)	0.062
**Race**, ***n*** **(%)**				
African American	3,525 (23.1)	3,453 (23.1)	72 (25.3)	0.80
Asian	529 (3.5)	519 (3.5)	10 (3.5)	
Caucasian	8,106 (53.2)	7,956 (53.2)	150 (52.6)	
Other/unknown	3,090 (20.3)	3,037 (20.3)	53 (18.6)	
**Ethnicity**, ***n*** **(%)**				
Hispanic	1,872 (12.3)	1,852 (12.4)	20 (7.0)	<0.001
Non-Hispanic	11,531 (75.6)	11,319 (75.6)	212 (74.4)	
Unknown	1,847 (12.1)	1,794 (12.0)	53 (18.6)	
**Region**, ***n*** **(%)**				
Midwest	5,531 (36.3)	5,457 (36.5)	74 (26.0)	<0.001
Northeast	5,521 (36.2)	5,372 (35.9)	149 (52.3)	
South	3,339 (21.9)	3,296 (22.0)	43 (15.1)	
West	520 (3.4)	507 (3.4)	13 (4.6)	
Other/unknown	339 (2.2)	333 (2.2)	6 (2.1)	
**Risk Factors**, ***n*** **(%)**				
Congestive heart failure	3,295 (21.6)	3,190 (21.3)	105 (36.8)	<0.001
Hypertension	10,962 (71.9)	10,718 (71.6)	244 (85.6)	<0.001
Diabetes	6,812 (44.7)	6,655 (44.5)	157 (55.1)	<0.001
Vascular disease	3,683 (24.2)	3,549 (23.7)	134 (47.0)	<0.001
Atrial fibrillation	2,731 (17.9)	2,634 (17.6)	97 (34.0)	<0.001
Smoke	4,180 (27.4)	4,091 (27.3)	89 (31.2)	0.14
**Labs at admission, median (IQR)**				
D-Dimer (μg/ml feu)	0.95 (0.56–1.83)	0.94 (0.55–1.81)	1.42 (0.76–3.96)	<0.001
C-reactive protein (mg/L)	93 (43–159)	93 (44–159)	85 (24–165)	0.24
Ferritin (ng/ml)	551 (250–1,153)	551 (250–1,152)	551 (235–1,343)	0.90
Lactate dehydrogenase (u/L)	343 (257–468)	343 (257–467)	361 (254–503)	0.13
Lymphocyte (× 10^9^/L)	1.00 (0.70–1.40)	1.00 (0.70–1.40)	0.90 (0.60–1.50)	0.71
Neutrophil (× 10^9^/L)	5.4 (3.7–8.2)	5.4 (3.7–8.2)	6.7 (4.2–9.6)	<0.001
Platelet count (× 10^9^/L)	212 (165–275)	212 (165–274)	213 (169–293)	0.33
White blood cell count (× 10^9^/L)	7.3 (5.4–10.3)	7.3 (5.4–10.2)	9.0 (6.4–12.2)	<0.001
**Medication administered**, ***n*** **(%)**				
Antiplatelet	5,231 (34.3)	5,012 (33.5)	219 (76.8)	<0.001
Anticoagulant	2,058 (13.5)	1,957 (13.1)	101 (35.4)	<0.001

**Figure 1:**
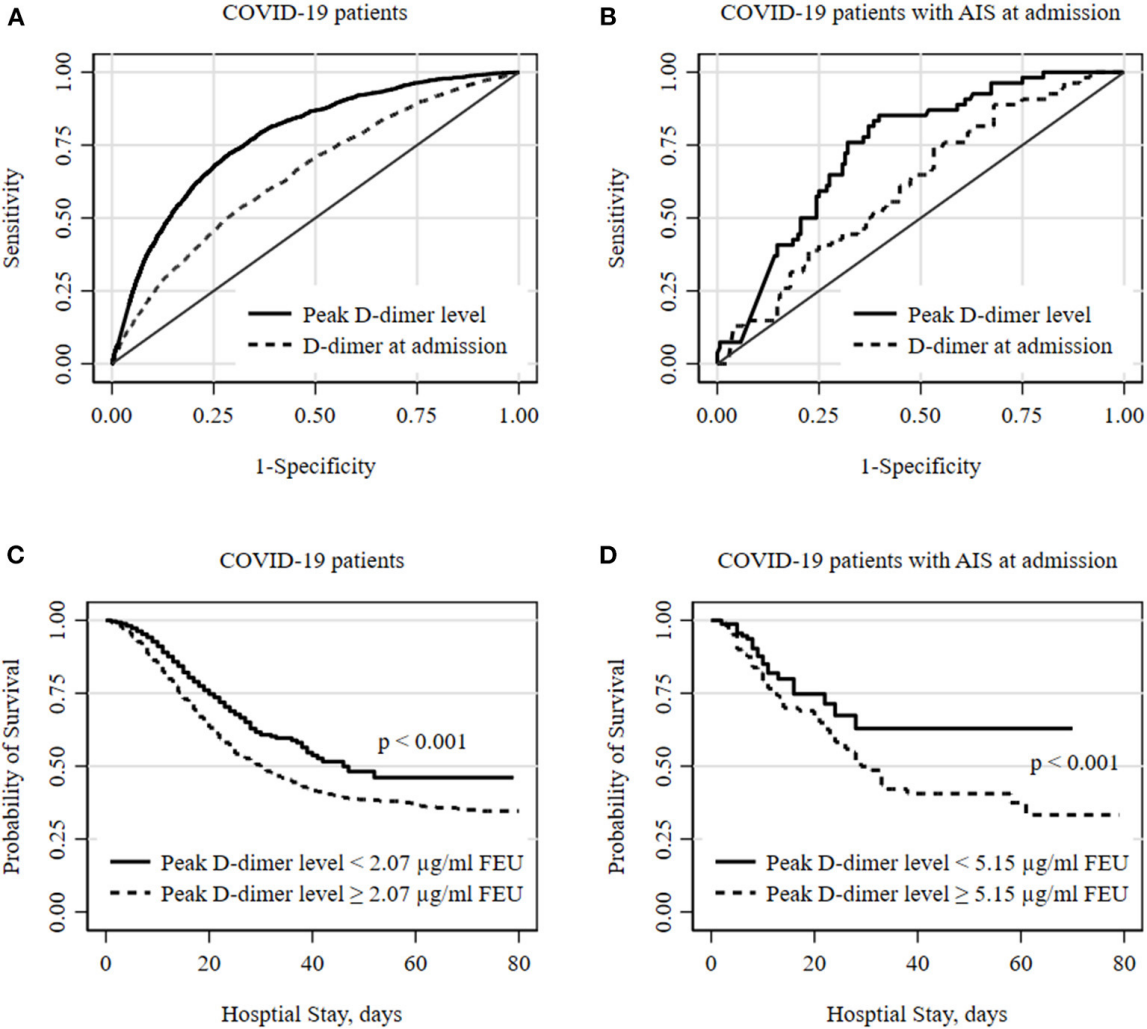
Optimal D-dimer levels to predict in-hospital mortality among COVID patients. **(A,B)** show receiver operator characteristic (ROC) curves for optimal D-dimer levels to predict deaths in all COVID-19 hospitalized patients and subgroup of patients with acute ischemic stroke (AIS) at admission. The peak D-dimer level performs better to predict deaths compared to D-dimer level at admission. The optimum cutoff thresholds of peak D-dimer levels were defined as the point on the ROC curve nearest to the upper left corner (0, 1) and were 2.07 μg/ml FEU with 72.3% sensitivity and 69.8% specificity for all and 5.15 μg/ml FEU with 72.6% sensitivity and 68.7% of specificity for AIS subgroup. **(C,D)** show Kaplan-Meier survival curves for all-cause death during hospital stay. Cutoff values of 2.07 and 5.15 estimated from ROC analyses were used for all and a subgroup of patients with AIS at admission, respectively. Statistical differences in survival curves between peak D-dimer levels below and above the cutoff values were assessed using a log-rank test.

The area under the ROC curve (C-statistic) using D-dimer levels at admission was 0.651 (95% CI, 0.637–0.664) for all COVID-positive patients and was 0.613 (95% CI, 0.529–0.697) for the AIS subgroup. Peak D-dimer levels performed better than admission level and were a good predictor for in-hospital mortality among all COVID-positive patients [c-statistic 0.774 (95% CI 0.764–0.784)] and among the AIS subgroup [c-statistic 0.751 (95% CI 0.691–0.810)] as shown in Figures 1A,B. The optimum cutoff values of peak D-dimer were identified as 2.07 μg/ml FEU with 72% sensitivity and 70% specificity for all COVID-positive patients and 5.15 μg/ml FEU with 73% sensitivity and 69% of specificity for the AIS subgroup. Kaplan-Meier survival curves constructed using these cutoff values show that patients with elevated peak D-dimer level above the cutoff value are less likely to survive both in all and the AIS subgroup (Figures 1C,D). Among all COVID-positive patients, elevated peak D-dimer level above the cutoff value was associated with increased mortality with crude RR 4.48 (95% CI, 4.12–4.87, *p* < 0.001) and adjusted RR 3.00 (95% CI, 2.75–3.28, *p* < 0.001) accounting for age, sex, race/ethnicity, and comorbidities. Among the AIS subgroup (Table 2), in-hospital mortality for those with elevated peak D-dimer level ≥ 5.15 μg/ml FEU was more than 3 times higher compared to those with below the cutoff D-dimer level [crude RR 3.44 (95% CI, 2.26–5.24, *p* < 0.001). After adjusting for covariates, we still found the elevated D-dimer level is associated with a significantly higher risk for death with adjusted RR 2.89 (95% CI, 1.87–4.47, *p* < 0.001)] in the AIS subgroup (Table 2). Increasing age and anti-coagulant use during the hospitalization were also associated with an increased of mortality among patients with AIS (Table 2).

**Table 2:** Factors associated with mortality among hospitalized COVID-19 patients with acute ischemic stroke.

****	**COVID 19 patients with AIS at admission (*****n*** **=** **285)**			
	**Crude RR (95% CI)**	***p*-value**	**Adjusted RR (95% CI)**	***p*-value**
Peak D-dimer ≥ 5.15 μg/ml feu)	3.44 (2.26–5.24)	<0.001	2.89 (1.87–4.47)	<0.001
**Age group**
18–44	1.00 (reference)		1.00 (reference)	
45–64	1.95 (0.50–7.60)	0.34	2.70 (0.88–8.32)	0.08
65–74	3.27 (0.86–12.41)	0.08	4.13 (1.38–12.40)	0.01
≥75	2.62 (0.69–9.96)	0.16	4.38 (1.45–13.22)	0.009
Male sex	1.12 (0.77–1.63)	0.54	0.87 (0.60–1.26)	0.47
**Race/ethnicity**
White	1.00 (reference)		1.00 (reference)	
Black	0.76 (0.44–1.31)	0.32	0.72 (0.45–1.16)	0.18
Hispanic	1.41 (0.77–2.59)	0.27	1.69 (0.95–3.00)	0.07
Other/unknown	1.24 (0.82–1.87)	0.32	1.09 (0.73–1.61)	0.68
**Risk factors**
Congestive heart failure	1.00 (0.69–1.46)	0.99	1.11 (0.76–1.60)	0.59
Hypertension	0.77 (0.49–1.21)	0.26	0.62 (0.38–1.01)	0.06
Diabetes	1.47 (1.00–2.15)	0.049	1.47 (1.01–2.16)	0.045
Vascular disease	1.18 (0.82–1.69)	0.36	1.05 (0.76–1.45)	0.77
Atrial fibrillation	1.32 (0.92–1.89)	0.13	1.06 (0.76–1.49)	0.73
Smoke	1.42 (0.99–2.04)	0.054	1.60 (1.14–2.24)	0.007
**Medication administered**
Antiplatelet	1.11 (0.71–1.72)	0.66	1.13 (0.76–1.69)	0.54
Anticoagulant	2.31 (1.62–3.31)	<0.001	1.60 (1.11–2.30)	0.01

## Discussion

In this study of a large multicenter database of patients with COVID positivity, patients presenting with AIS had greater D-dimer levels compared to those without AIS, and thresholds to predict mortality were higher in the AIS population. In patients with AIS, peak values above 5.15 μg/ml FEU were associated with a nearly three-fold risk of in-hospital mortality.

A pro-inflammatory hypercoagulable state has been well-associated with the COVID-19 infection (11, 12). Elevated D-dimer levels have been found in COVID-19 patients with coagulopathy and several observational studies reported elevated D-dimer level was a good predictor of ICU admission or in-hospital death (4, 6, 13). Independently, D-dimer has been previously identified as a biomarker for AIS and associated with stroke severity (9, 14). Therefore, the prognostic value of D-dimer in COVID-19 could differ for COVID-19 patients presenting with AIS, in whom D-dimer levels may be independently elevated. Our study confirmed that D-dimer levels at admission were elevated among COVID-19 patients [0.95 (0.56–1.83) μg/ml FEU] beyond normal range (< 0.5 μg/ml FEU) and greater elevations were observed among COVID-19 patients presenting with AIS [1.42 (0.76–3.96) μg/ml FEU] (15, 16).

We found the optimal cutoff values to predict mortality in COVID-19 patients were 2.07 μg/ml FEU with 72.3% sensitivity and 69.8% specificity for all. The cutoff value of 2.07 μg/ml FEU for all hospitalized COVID-19 patients is similar to previous findings. Zhang et al. reported an optimum cutoff value of D-dimer as 2.0 mg/ml within 24 h of hospital admission and Yao et al. reported D-dimer levels > 2.14 mg/ml on admission as a predictor of mortality (4, 5). However, most of these studies used the level of D-dimer on admission only and few studies discussed changes in D-dimer levels over time and showed an association between dynamic changes of D-dimer level with the prognosis of COVID-19 (11, 17). In our study, peak D-dimer levels performed better than admission level in predicting in-hospital mortality among all COVID-19 patients as well as the AIS subgroup. Since the time from COVID-19 onset to hospitalization varies across different patient characteristics and health care systems, the peak level reflects better dynamic changes of patient's progress and be more uniformed to be used than the D-dimer level on admission. Soni et al. also tested with both D-dimer levels on admission and with peak value during the hospital stay and found the peak level performs better and reported the cutoff value of 2.01 mg/ml with a sensitivity of 73.3% and a specificity of 70.0%, with a C-index of 0.789 (6). Importantly, the cutoff value for COVID-19 patients presenting with AIS was more than twice as high as the cutoff value for non-AIS patients, reflecting a greater elevation of D-dimer levels among AIS patients.

We assessed other lab values including inflammatory markers but found they were not significantly different between stroke and non-stroke COVID-19 patients except neutrophil and white blood cell counts. We also tested their optimal cutoff values and found they had similar or lower performance in predicting hospital mortality among all COVID-19 patients and the AIS subgroup. It is also worth noting that we observed an increased likelihood of mortality in AIS COVID-19 positive patients with increasing age and with anticoagulant use. The increased mortality associated with anticoagulant use may be secondary to increased usage in patients with more severe strokes or extensive thrombosis.

Our study has several limitations. Unlike single provider-based datasets, this multicenter database contained variations in D-dimer units across different hospitals, and as a result, we converted the reporting units to μg/ml FEU. In addition, our dataset contained limited descriptions of stroke subtypes and severity, precluding additional subgroup analyses. However, despite the potential heterogeneity and limited information, we found similar cutoff values compared to previous studies. Since we used a large EHR dataset covering patients across the country, we believe our study provides the external validity of the established cutoff value and presents the feasibility of conducting reliable observational studies using EHR data.

## Conclusion

COVID-19 patients with AIS present with higher D-dimer levels compared to those without AIS. D-dimer functions well as a predictor of mortality in this subgroup, however the threshold for predicting this outcome is substantially greater.

## Data Availability Statement

The data analyzed in this study was obtained from Optum, the following licenses/restrictions apply: the data that support the findings of this study are available from Optum upon reasonable request. Requests to access these datasets should be directed to Sunil A. Sheth, ssheth@post.harvard.edu.

## Ethics Statement

The studies involving human participants were reviewed and approved by the Committee for the Protection of Human Subjects (CPHS) at The University of Texas Health Science Center at Houston. Written informed consent for participation was not required for this study in accordance with the national legislation and the institutional requirements.

## Author Contributions

YK, SK, and SS drafted the manuscript. All authors listed have made a substantial, direct and intellectual contribution to the work, and approved it for publication.

## Conflict of Interest

The authors declare that the research was conducted in the absence of any commercial or financial relationships that could be construed as a potential conflict of interest.
